# Fosfomycin Addition to Poly(_D,L_-Lactide) Coating Does Not Affect Prophylaxis Efficacy in Rat Implant-Related Infection Model, But That of Gentamicin Does

**DOI:** 10.1371/journal.pone.0165544

**Published:** 2016-11-02

**Authors:** Anil Gulcu, Alp Akman, Ahmet Fahir Demirkan, Ali Cagdas Yorukoglu, Ilknur Kaleli, Ferda Bir

**Affiliations:** 1 Orthopedics and Traumatology Department, Faculty of Medicine, Pamukkale University, Denizli, Turkey; 2 Microbiology Department, Faculty of Medicine, Pamukkale University, Denizli, Turkey; 3 Pathology Department, Faculty of Medicine, Pamukkale University, Denizli, Turkey; Cambridge University, UNITED KINGDOM

## Abstract

Gentamicin is the preferred antimicrobial agent used in implant coating for the prevention of implant-related infections (IRI). However, the present heavy local and systemic administration of gentamicin can lead to increased resistance, which has made its future use uncertain, together with related preventive technologies. Fosfomycin is an alternative antimicrobial agent that lacks the cross-resistance presented by other classes of antibiotics. We evaluated the efficacy of prophylaxis of 10% fosfomycin-containing poly(_D_,_L_-lactide) (PDL) coated K-wires in a rat IRI model and compared it with uncoated (Control 1), PDL-coated (Control 2), and 10% gentamicin-containing PDL-coated groups with a single layer of coating. Stainless steel K-wires were implanted and methicillin-resistant *Staphylococcus aureus* (ATCC 43300) suspensions (10^3^ CFU/10 μl) were injected into a cavity in the left tibiae. Thereafter, K-wires were removed and cultured in tryptic soy broth and then 5% sheep blood agar mediums. Sliced sections were removed from the tibiae, stained with hematoxylin-eosin, and semi-quantitatively evaluated with X-rays. The addition of fosfomycin into PDL did not affect the X-ray and histopathological evaluation scores; however, the addition of gentamicin lowered them. The addition of gentamicin showed a protective effect after the 28^th^ day of X-ray evaluations. PDL-only coating provided no protection, while adding fosfomycin to PDL offered a 20% level protection and adding gentamicin offered 80%. Furthermore, there were 10^3^ CFU level growths in the gentamicin-added group, while the other groups had 10^5^. Thus, the addition of fosfomycin to PDL does not affect the efficacy of prophylaxis, but the addition of gentamicin does. We therefore do not advise the use of fosfomycin as a single antimicrobial agent in coating for IRI prophylaxis.

## Introduction

Implant-related infections (IRI) are most commonly seen as a serious complication if there is foreign material [1]. It is very difficult to treat due to the formation of biofilms, which decreases their susceptibility to antibiotics [2–8]. Thus, prevention may be the best form of treatment.

Gentamicin-added poly(_D_,_L_-lactide) (PDL) coating has been extensively studied in vitro [9–11] and in vivo [11–14] for the prevention of IRI. Preliminary reports for a coated tibial intramedullary nail show favorable characteristics and effectiveness [14,15]. The present heavy local and systemic administration of gentamicin can lead to increased resistance, which has made its future use uncertain, together with related preventive technologies. [16]. By using it in prophylaxis, the efficacy of the antibiotic may be lost if it is required in a further infection. Fosfomycin may be an alternative to gentamicin as it lacks the cross-resistance that other classes of antibiotics present because it acts on synthetic steps unaffected by other agents [17]. It is a broad-spectrum antibiotic and is effective against methicillin-resistant *Staphylococcus aureus* (MRSA) [18]. Additionally, it has superior osseophilic behavior in contrast with other antibiotic agents, which may be explained by the similarity in its structure to hydroxyapatite [19,20]. Therefore, it may be beneficial for the treatment of osseous infections. Furthermore, using fosfomycin in prophylaxis allows the use of aminoglycosides to treat further infections.

This study evaluated the efficacy of fosfomycin added to a PDL coating in a rat IRI model, and compared it to a gentamicin-added PDL coating, PDL-only coating, and bare implant.

## Materials and Methods

The study was approved by the Experimental Animal Research Ethics Committee of Pamukkale University, Denizli, Turkey (Reg. No.: 60758568-020/58916, Reg. Date: 17/04/2014). The study was performed in the Experimental Research Unit in Pamukkale University. The rats were supplied with unlimited tap water (ad libitum) and given standard rodent feed. Animals were kept in sufficiently large cages at a constant temperature of 22°C with a 12/12 h light/dark cycle. All applicable international, national, and/or institutional guidelines for the care and use of animals were followed. All procedures performed in studies involving animals were in accordance with the ethical standards of the institution or practice at which the studies were conducted.

The IRI rat model was conducted according to a previously reported protocol [12], with some modifications. The pathogen used was MRSA (ATCC 43300). We evaluated the strain using an Automated Microbiology System (BD Phoenix Automated Microbiology System, Becton Dickinson, NJ, USA), which was found to be resistant to penicillin, oxacillin, clindamycin, and erythromycin, but sensitive to vancomycin, teicoplanin, linezolid, daptomycin, nitrofurantoin, and gentamicin. The strain was found to be susceptible to fosfomycin using the disc diffusion method [21] (i.e., 48 mm zone of diameter), which is in concordance with previous reports using the same method [22,23]. (S1 Fig)

**Fig 1:**
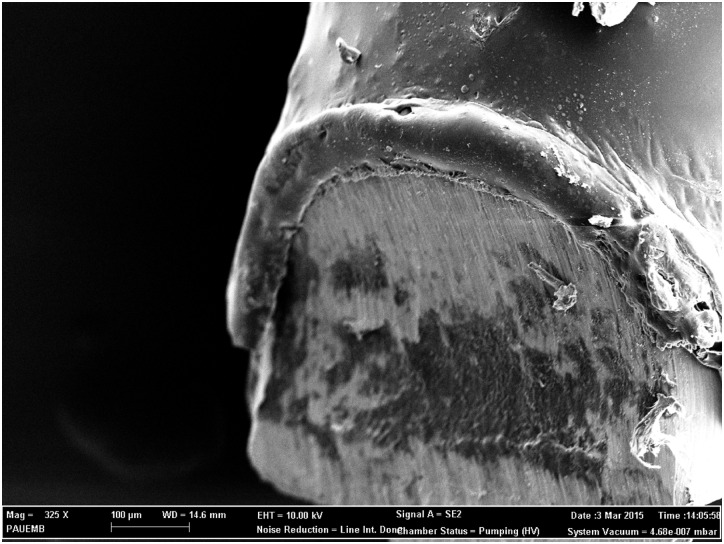
Electron microscopy image at 325x magnification showing the poly(_D_,_L_-lactide) (PDL) coating.

PDL (Boehringer Ingelheim, Ingelheim, Germany) was chosen as the biodegradable coating polymer and drug carrier. PDL was dissolved in 1.5 ml of volatile solvent (Chloroform; Sigma-Aldrich, St. Louis, USA) at room temperature. Lyophilized gentamicin sulfate (80 mg/2 ml, generic) or fosfomycin sodium (Ventranal; Sigma-Aldrich, St. Louis, USA) was suspended in the solution and added according to each groups need, yielding a concentration of 10% gentamicin or fosfomycin at the base weight to weight (w/w) of the polymer. The solution was sterile filtered. Sterilized stainless steel (S-steel) K-wires 0.8 mm in diameter were dipped into the solution and dried under sterile laminar airflow conditions. The coating process was done only once (Fig 1). K-wires were kept in sterile conditions until implantation.

Forty female, 3-month-old Wistar albino rats with averaging weight of 250 g were used in the study. The rats were randomly assigned to one of four groups:

Group 1: Uncoated group (Control 1),
Group 2: PDL-only coated group (Control 2),
Group 3: Gentamicin-added PDL coated group, and
Group 4: Fosfomycin-added PDL coated group.

**Fig 2:**
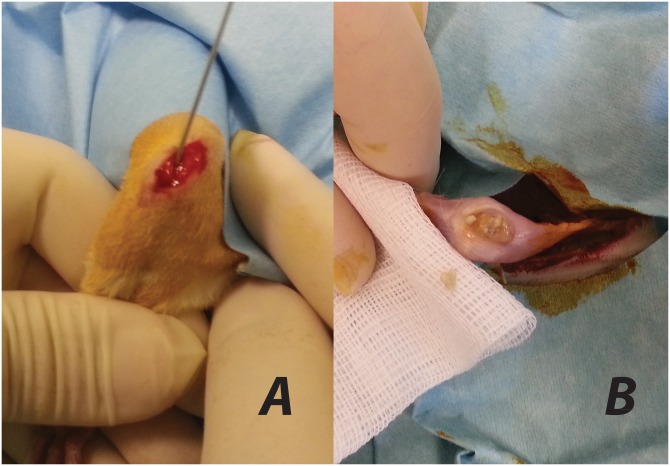
Implantation. (a) Implantation of K-wire into rat tibias and (b) a view of the entrance site of K-wire after being sacrificed.

Surgery was performed under general anesthesia by a weight-adopted intraperitoneal injection of 2% xylazine (12 mg/kg) and ketamine hydrochloride (80 mg/kg). The left hind leg was shaved, and disinfected with 10% povidone-iodine solution. The animals were placed on sterile drapes, the bodies were covered, except the left hind leg, with sterile sheets, and the left hind foot was draped separately. Skin and fascia at the proximal tibial metaphysis were incised (5 mm in length). With a hand-driven burr, a 1 mm diameter K-wire was drilled through the cortical and cancellous bone to access the medullary cavity of the proximal metaphysis. The medullary cavity was bluntly reamed with K-wire in order to reach the narrowing distal part of the tibial cavity. After removal, 10 μl of bacterial suspension containing 10^3^ CFU/10 μl MRSA was injected with a 50 μl micro syringe for contamination of the medullary cavity. A suitable K-wire was inserted into intramedullary cavity (Fig 2a). The excess parts of the K-wires were cut off at the site of entry. Soft tissue was irrigated, and skin and fascia were interruptedly sutured with a single knot technique.

Animals were followed up for 42 days and then sacrificed. All procedures were carried out with intraperitoneal anesthesia. On the day of surgery and regularly throughout the observation period, the body weight and body temperature of rats were measured. The clinical conditions of the animals were checked and X-rays of the left tibia in two planes were performed. On days 1, 3, 7, 14, 21, 28, 35, and 42, their rectal body temperature was measured using a digital thermometer and recorded as Centigrade Celsius (°C), and body weight was determined using a precision scale and recorded as grams. Animals were inspected for clinical signs of infection (appearance of wound and soft tissue at entry site of implant, joint effusion, loss of mobility, general condition). On days 1, 14, and 42, blood samples (1 to 2 ml) were taken from the right atriums and evaluated for the hemoglobin (Hb) level (g/dl) and white blood cell (WBC) count (1000/μl).

**Fig 3:**
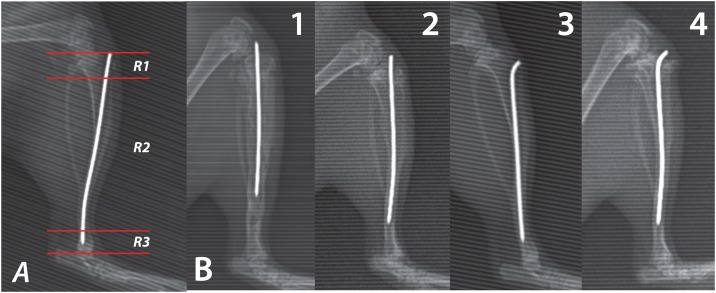
X-ray evaluation. (a) Illustration showing the region of interests in a lateral X-ray (R1: Proximal epi-/metaphysis, R2: Diaphysis and R3: Distal epi-/metaphysis), and (b) different levels of X-ray finding in four different subjects. (Note: Numbers denote the subject’s group).

X-rays of the anterior-posterior and lateral views were performed on days 1, 7, 14, 28, 35, and 42, with a distance from focus to film of 110 cm. A digital X- ray unit is used to assess the development and progression of bone infection. The following three regions of interest (ROI) were determined and separately evaluated (Fig 3): (R1) proximal epi-/metaphysis, (R2) diaphysis, and (R3) distal epi-/metaphysis. The following radiographic appearances were assessed according to a modified score by An and Friedman [24]: (1) Periosteal reaction, (2) Osteolysis, (3) Soft tissue swelling, (4) Deformity, (5) General impression, (6) Spontaneous fracture, and (7) Sequestrum formation. Parameters 1 to 5 ranged from 0 (absent), 1 (mild), 2 (moderate) to 3 (severe) and were evaluated for every ROI. Parameters 6 and 7 were judged as 0 (absent) or 1 (present). For assessment of parameters 6 and 7, radiographs were not subdivided into ROI. All radiographs were scored in comparison to the 1^st^ day radiographs. The maximum score achievable was 47.

**Fig 4:**
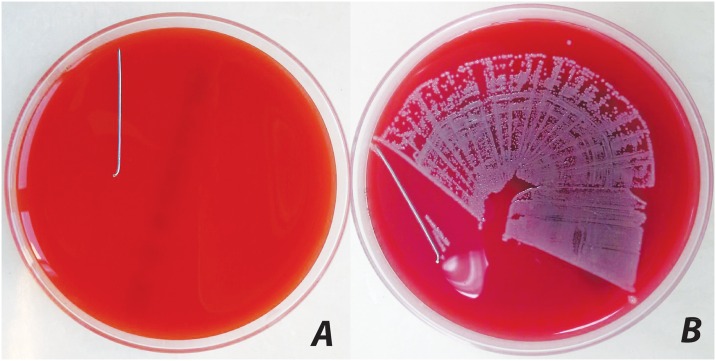
Bacterial growth on 5% sheep blood agar after implant rolling in demonstration samples. (a) No bacterial growth and (b) bacterial growth.

On day 42, under intraperitoneal anesthesia, the chest cavity was opened. Three milliliters of blood was drawn from the right atrium and put into an aerobic culture medium (BD BACTEC FX; Becton Dickinson, Maryland, USA). Then, a lethal dose of potassium chloride was applied by intracardiac injection. The tibiae of the left hind legs were dissected under sterile conditions using separate instruments for preparation of the skin and the tissue underneath (Fig 2b). A swapping culture was taken from the K-wire entrance with a transport swap. The entire soft tissue was removed. The K-wires were placed in 2.5 ml of sterile TSB mediums after sonofication and incubated at 37°C for 48 h. The suspensions were subsequently diluted (1:1, 1:10, and 1:100) and 10 μl of samples were streaked on 5% sheep blood agar (SBA) plates and incubated at 37°C for 48 h. After that, the CFUs on SBA plates were counted visually and bacterial growth in the TSB was judged (cloudy: positive growth; clear: no growth). Bacteria isolated from cultures were evaluated by a catalase test, plasma coagulase test, gram staining, and automated microbiology system for the identification of contamination and evaluation of resistance. There were no contaminating *S*. *aureus* species in this study as very strict isolating rules were applied (Fig 4).

**Fig 5:**
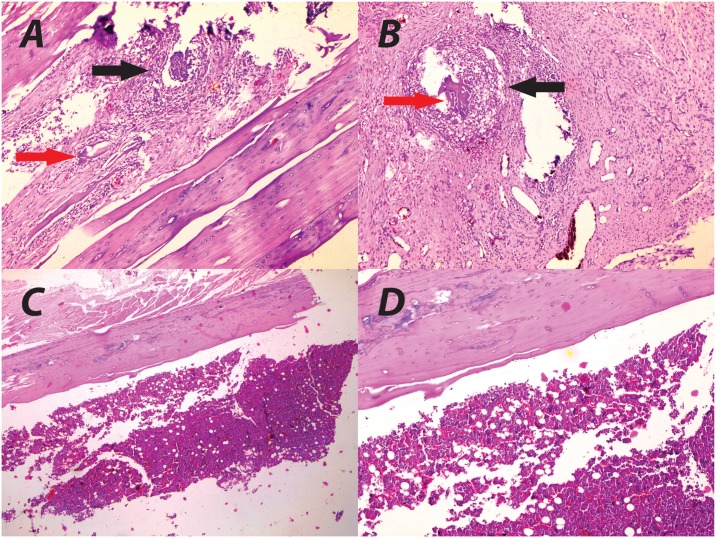
Histopathological evaluation. Abscess (black arrow) and sequestrum (red arrow) formation (a and b) compared with normal histology (c and d) in hematoxylin-eosin (H&E) staining, with x10 and x20 magnification, respectively.

Longitudinal sections 5 μm thick were cut by a microtome at the sagittal plane. Slices were stained with hematoxylin-eosin (H&E) (Sigma-Aldrich, St. Louis, USA). Slices of the same bone plane were evaluated for the following parameters, according to a modified score of Petty et al. [25]: (1) Abscess formation, (2) Sequestrum formation, (3) Cortical enlargement, and (4) Cortical destruction. Parameters were scored semi-quantitatively as: 0 (absent), 1 (mild), 2 (moderate) and 3 (present). All parameters were assessed for each ROI. The maximum score achievable was 12 (Fig 5).

SPSS v22 (IBM, New York, USA) was used for statistical evaluation. A non-parametric Kruskal-Wallis Test was used for independent group comparisons, whereas a Friedman test was used for dependent groups. Data were analyzed within 95% confidence intervals and a P value less than 0.05 was considered statistically significant.

## Results

Osteomyelitis was established in all subjects of control groups. Mean body weights, body temperatures, Hb, and WBC counts did not significantly change at the follow-up time in all groups.

**Fig 6:**
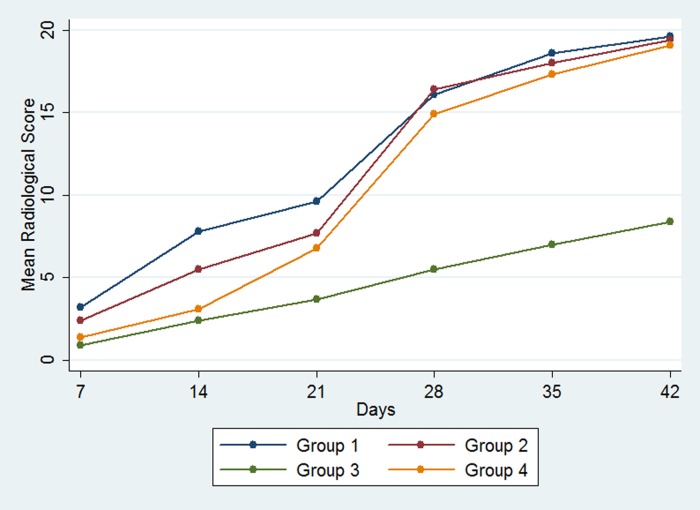
Changes in the mean radiological scores for groups by time.

From the X-ray evaluations, Group 4 shows significantly lower scores than Group 1 on the 14^th^ day and 21^st^ day (p < 0.001 and p = 0.013 respectively), but not after or before these days. Group 4 did not show significantly lower scores than Group 2 or higher scores than Group 3 in all evaluations. In contrast, Group 3 showed significantly lower scores than Group 1 on the 7^th^ day (p = 0.003), and Group 1 and 2 on the 14^th^ day (p < 0.001 and p = 0.005, respectively) and 21^st^ day (p < 0.001 and p = 0.004 respectively), but not after. Group 3 showed significantly lower scores than Groups 1, 2, and 4 on the 28^th^ day (p < 0.001, p < 0.001, and p = 0.004, respectively), 35^th^ day (p < 0.001, p = 0.001, and p = 0.003, respectively), and 42^nd^ day (p < 0.001, p = 0.001, and p = 0.001, respectively). Thus, the addition of fosfomycin to PDL did not alter X-ray scores, but the addition of gentamicin did lower the scores. The resultant effects of the addition of gentamicin started to show from the 28^th^ day (Fig 6).

From the histopathological evaluation, Group 3 showed significantly lower scores compared to Group 1 or Group 2 but not Group 4 (p = 0.004, p = 0.049, and p = 0.240, respectively). In contrast, Group 4 showed lower scores than Group 1 or 2, but these values did not reach a statistically significant level. Thus, the addition of fosfomycin to PDL showed no difference according to the histopathological scoring, but the addition of gentamicin reduced the scores compared to the controls (Table 1).

**Table 1:** Histopathological and microbiological evaluation results.

	Medium	Group 1	Group 2	Group 3	Group 4
Histopathological score¶	H&E	10.2 ± 1.6 (9–12)	9 ± 1.6 (7–11)	2.4 ± 1.1 (1–4)	8.2 ± 1.3 (7–10)
Microbiological results#	SBA	10* (100%)	10* (100%)	2** (20%)	8* (80%)
	TSB	10 (100%)	10 (100%)	2 (20%)	8 (80%)

^¶^Values are given in form of mean ± standard deviation (minimum—maximum).

^#^Values are given in the form of infected medium number in the group (ratio %).

*Over 10^5^ CFU level growth,

**10^3^ CFU level growth.

There was no bacterial growth on the K-wire entrance swapping cultures or blood cultures. Over 10^5^ CFU level bacterial growths were observed in all samples from Groups 1 and 2 in SBA cultures. The same level of bacterial growth was observed in 8/10 samples in Group 4. In contrast, there were 10^3^ CFU level growths in 2/10 samples from Group 3. The TSB mediums were cloudy in all samples from Groups 1 and 2, 8/10 in Group 4, and 2/10 in Group 3, however the rest of the mediums were clear. Thus, the bacterial growths in TSB mediums are in concordance with the culture swapping results. PDL-only coating provided no protection. From Table 1, the microbiological evaluation shows that the addition of fosfomycin to PDL gave a 20% level protection whilst the addition of gentamicin gave 80%.

## Discussion

*S*. *aureus* is the most common cause of osteomyelitis, whether implant-related or not [26]. Direct application of potential antimicrobial agents to the tissue-implant interface may provide better infection prophylaxis. Coating the implant surface with antimicrobial peptides can reduce the adhesion of bacteria and biofilm formation, and therefore have a potential protective role in IRI [27]. As an alternative drug for gentamicin, fosfomycin shows antimicrobial activity against biofilms, particularly in combination with fluoroquinolones or aminoglycosides against gram-negative bacteria [28–31]. Fosfomycin can penetrate multilayer biofilms and studies suggest that the penetration into the cells occurs in the low growth rate phase [30]. Fosfomycin can also disintegrate biofilms to enhance the permeability of other antibiotics, as seen with studies on gram-negative bacteria [32]. However, there is controversy over whether this occurs in MRSA [23].

A thin layer of PDL can be used as carrier of many kinds of bioactive agents due to its biodegradability [33–39]. It is highly biocompatible to bone tissue and does not interfere with bone healing processes [40,41]. Studies on the release kinetics of PDL found that bioactive agents are released rapidly with an initial burst and followed by a slow release of up to 14 to 16 weeks [9,11,39,42]. However, the release kinetics changes if there is a thick layer of polymer. An in vitro study found that the entirety of gentamicin is released if one layer of PDL coating is applied, whereas only one-third of the drug remained if the coating was four layers thick [9].

A research group from Germany extensively studied the protection potential of gentamicin-added PDL coating against MRSA. In their first study, they compared 10% w/w gentamicin-containing PDL coated titanium K-wires with uncoated and PDL-only coatings using a rat model of IRI (MRSA with 10^3^ CFU IS). The addition of gentamicin achieved a 30% level protection [12]. In their second study, using same model but with lower inoculum size (IS) (10^2^), they compared the same coated implant with systemically applied gentamicin (4 mg/kg, single shot prior to surgery) and combination of systemic and local application. They found that for prevention, local application is better than systemic, and the protection ratios were 90%, 15%, and 80%, respectively [13]. In their third study, they compared the same groups in their first study with a placebo, and compared systemic application to local application. They found that systemic application provides 15% protection, whereas local application provides 85% [14]. The systemic usage in their experiments was very short lived (i.e. single shot) compared to the local usage, due to the drug release profile of the polymer, and thus should be interpreted accordingly. Our results (80% protection ratio with gentamicin) are in accordance with previous studies (including our reference model study), despite the higher IS of our study (10^3^). Additionally, we used an S-steel implant, to which *S*. *aureus* adheres to better [43,44].

Several studies on the treatment of IRI in animal models induced by MRSA inoculation have compared various regiments of systemic antibiotic application. A study using a rat osteomyelitis model found that vancomycin provides no protection, however, fosfomycin therapy is 100% protective (10^8^ CFU IS) [34]. In a tissue-cage model using rats (10^5^ CFU IS), the combination of daptomycin-rifampicin showed the highest protection ratio (94%) compared to daptomycin-rifampicin and fosfomycin-rifampicin (82% and 79%, respectively) [45]. In another tissue-cage model using guinea pigs (10^6^ CFU IS), the most effective therapy was found to be a combination of fosfomycin-rifampicin (83%) followed by daptomycin-rifampicin (67%) [23].

Concerning local application, one study examined the effects of fosfomycin-rifampicin in combination in an IRI model using ink-jet coating technology [46]. Coated or uncoated S-steel K-wires were inserted into the rabbit tibiae, which were contaminated with 10^5^ or 10^6^ CFU IS of methicillin-sensitive or -resistant *S*. *aureus*. Rifampicin-fosfomycin coating showed excellent antibacterial activity (100%) compared to the controls (0%). However, fosfomycin monotherapy was not included in their study. Furthermore, their work did not include a carrier agent, which alters the release kinetics.

Our results indicate that adding fosfomycin to the PDL coating makes has a minimal impact on the protection ratio, as we found a 20% ratio with fosfomycin but 80% with gentamicin. Furthermore, there were 10^3^ CFU level growths in two samples of the gentamicin-added group, whereas the other groups had 10^5^ CFU level growths. Our results also question the anti-biofilm effect of fosfomycin on MRSA.

One limitation of our study is the limited number of samples, with only 10 subjects per group. As such, the statistical power of our study was limited. The comparison of different ISs (10^2^ and 10^6^ CFU) would improve our understanding of the effect of adding fosfomycin to PDL and therefore improve any potential advice given to clinicians, based on the severity of the infection. We could have been used more fosfomycin dosage, but it should be remembered that higher antibiotic concentrations may impair the integrity of the polymer and the use of multiple layer coatings may lead to some unreleased drug. Additionally, no pharmacokinetic/pharmacodynamics evaluations were performed in this study nor any additional procedures to detect small colony variants or resistance development. Therefore, we consider this a pilot study. Lastly, the differences in responses to infections in the different groups should be considered carefully, as the body weights of subjects in the different groups were not paired.

In summary, we found that the addition of fosfomycin to PDL makes no difference to prophylaxis efficacy, however gentamicin does. Thus, fosfomycin should not be used as a single antimicrobial agent in PDL coating for IRI prophylaxis. Further research is required to elucidate the ineffectiveness of fosfomycin.

## Supporting Information

S1 Fig Disc-diffusion test of *Staphylococcus aureus* against fosfomycin. A 48 mm zone of diameter was found.
(TIF)

S2 Fig Electron microscopy image at 54x magnification showing the poly(_D_,_L_-lactide) (PDL) coating. (TIF)

S3 Fig Electron microscopy image at 325x magnification showing the poly(_D_,_L_-lactide) (PDL) coating with thickness measurements. (TIF)

S4 Fig The extracted tibiae of the left hind legs. (TIF)

S5 Fig Inoculation from tryptic soy broth to %5 sheep blood agar. (TIF)
