# Supplementary figures and images for: Fosfomycin Addition to Poly(D,L-Lactide) Coating Does Not Affect Prophylaxis Efficacy in Rat Implant-Related Infection Model, But That of Gentamicin Does
Source: PLoS One. 2016 Nov 2;11(11):e0165544. doi: 10.1371/journal.pone.0165544 (PMC5091905; doi:10.1371/journal.pone.0165544)

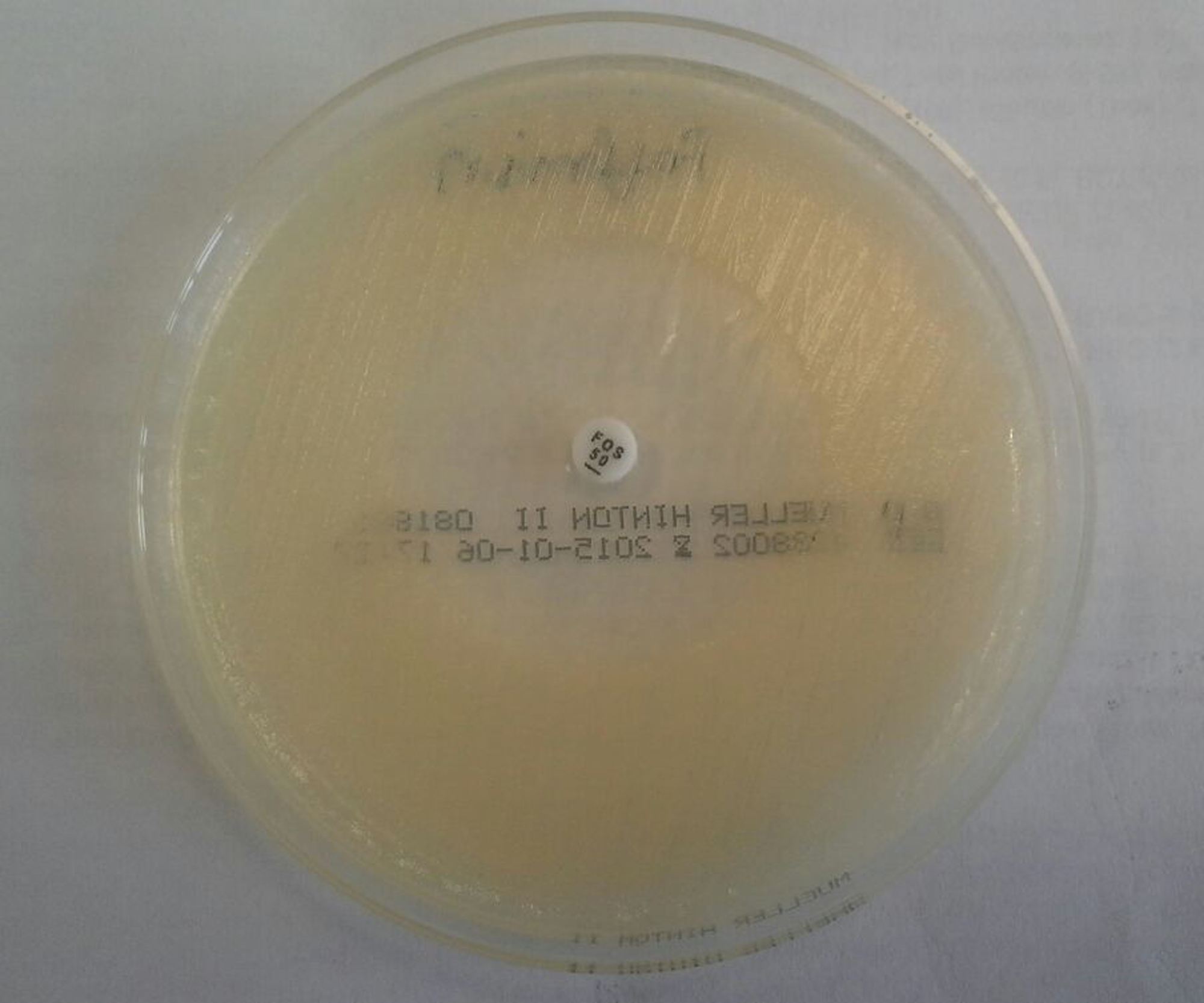

Supplement: S1 Fig — A 48 mm zone of diameter was found. (TIF) [file pone.0165544.s001.tif]

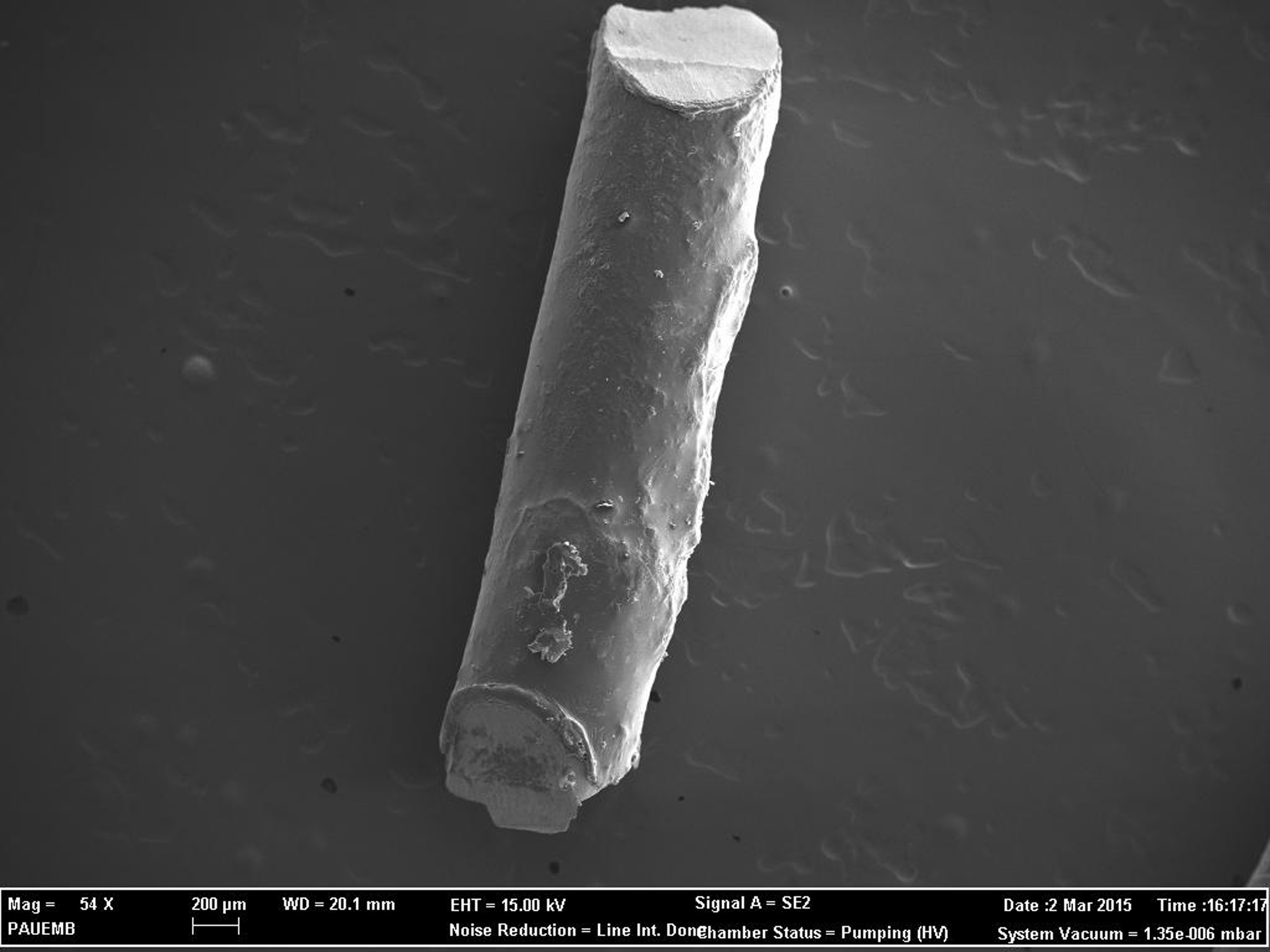

Supplement: S2 Fig — (TIF) [file pone.0165544.s002.tif]

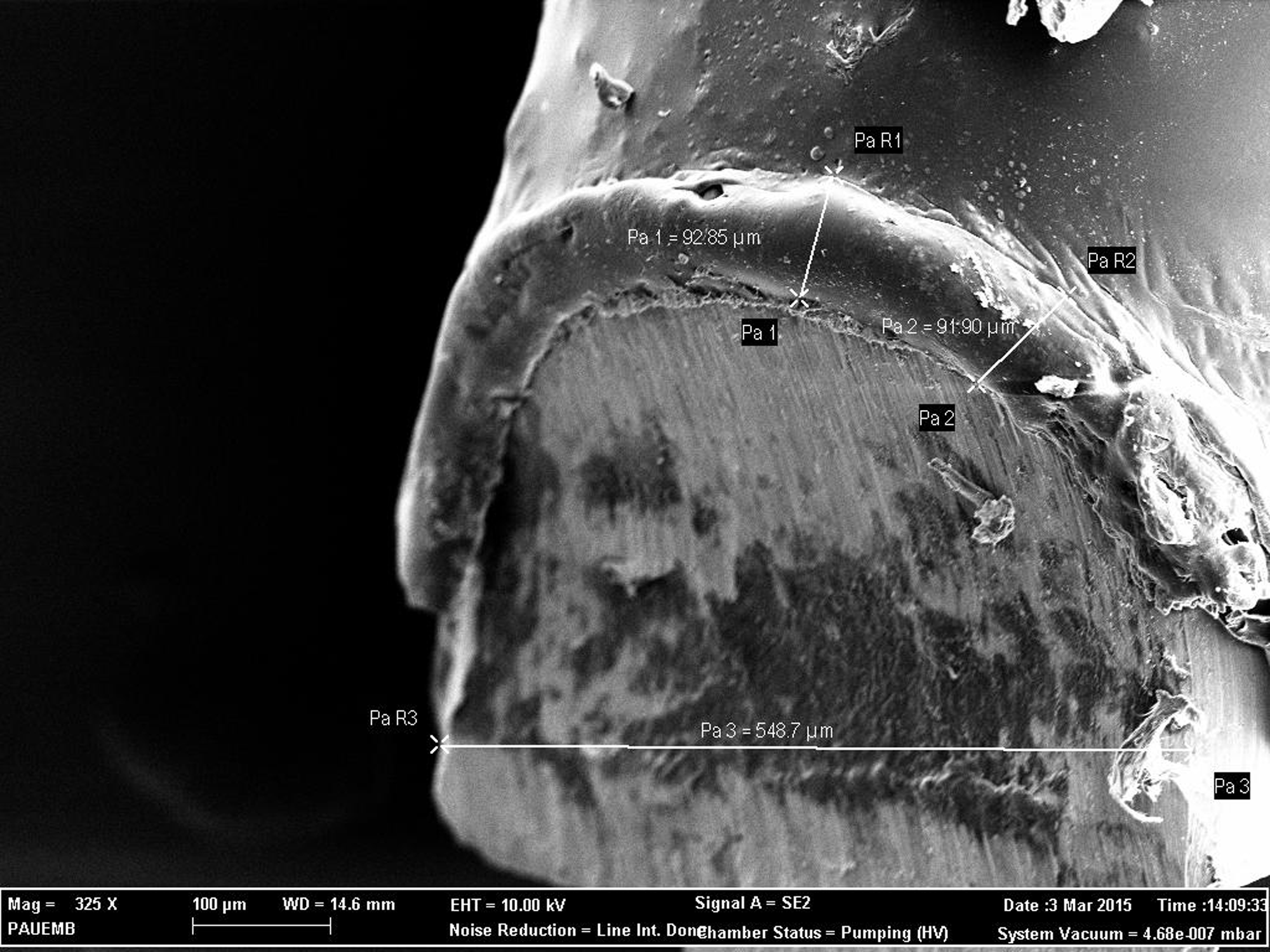

Supplement: S3 Fig — (TIF) [file pone.0165544.s003.tif]

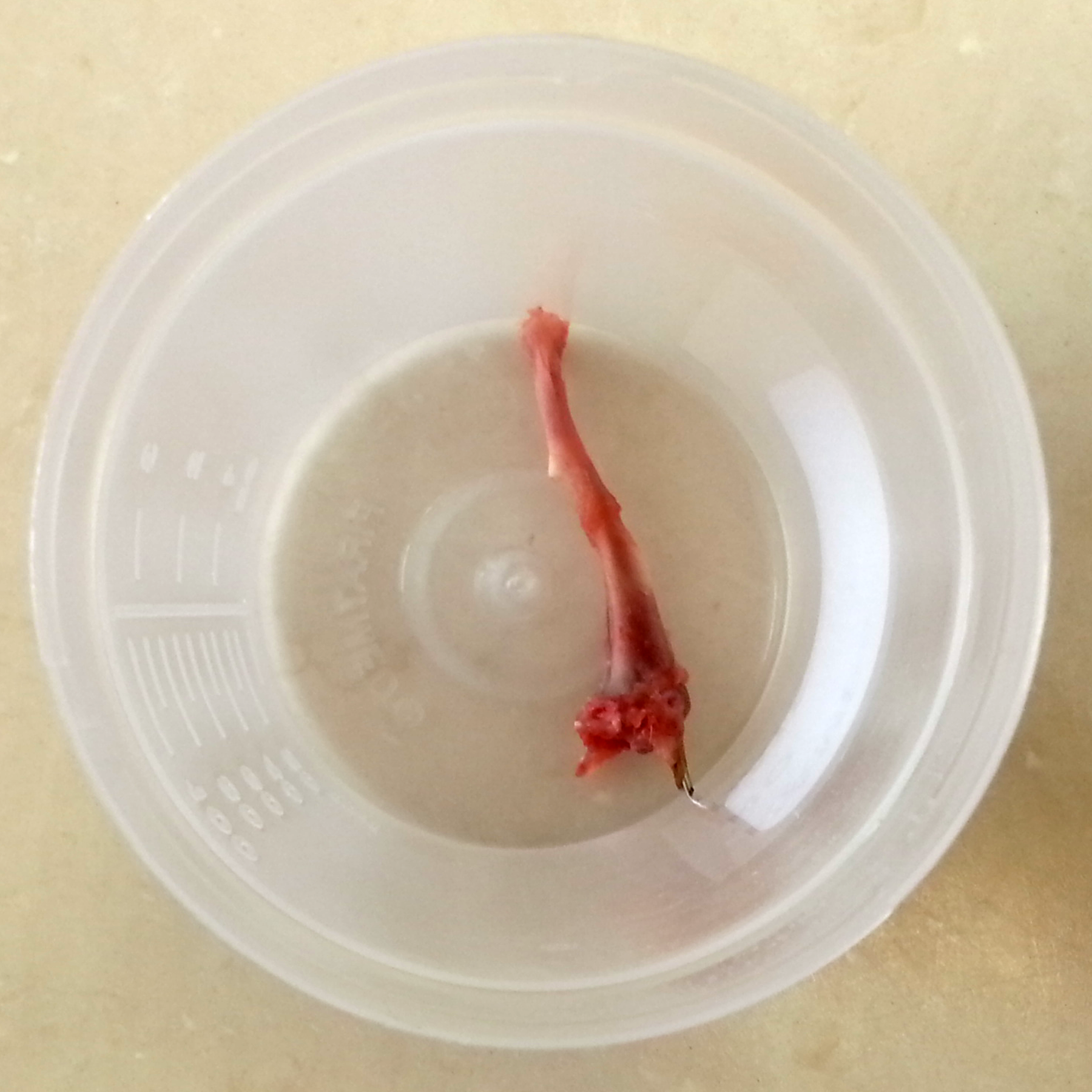

Supplement: S4 Fig — (TIF) [file pone.0165544.s004.tif]

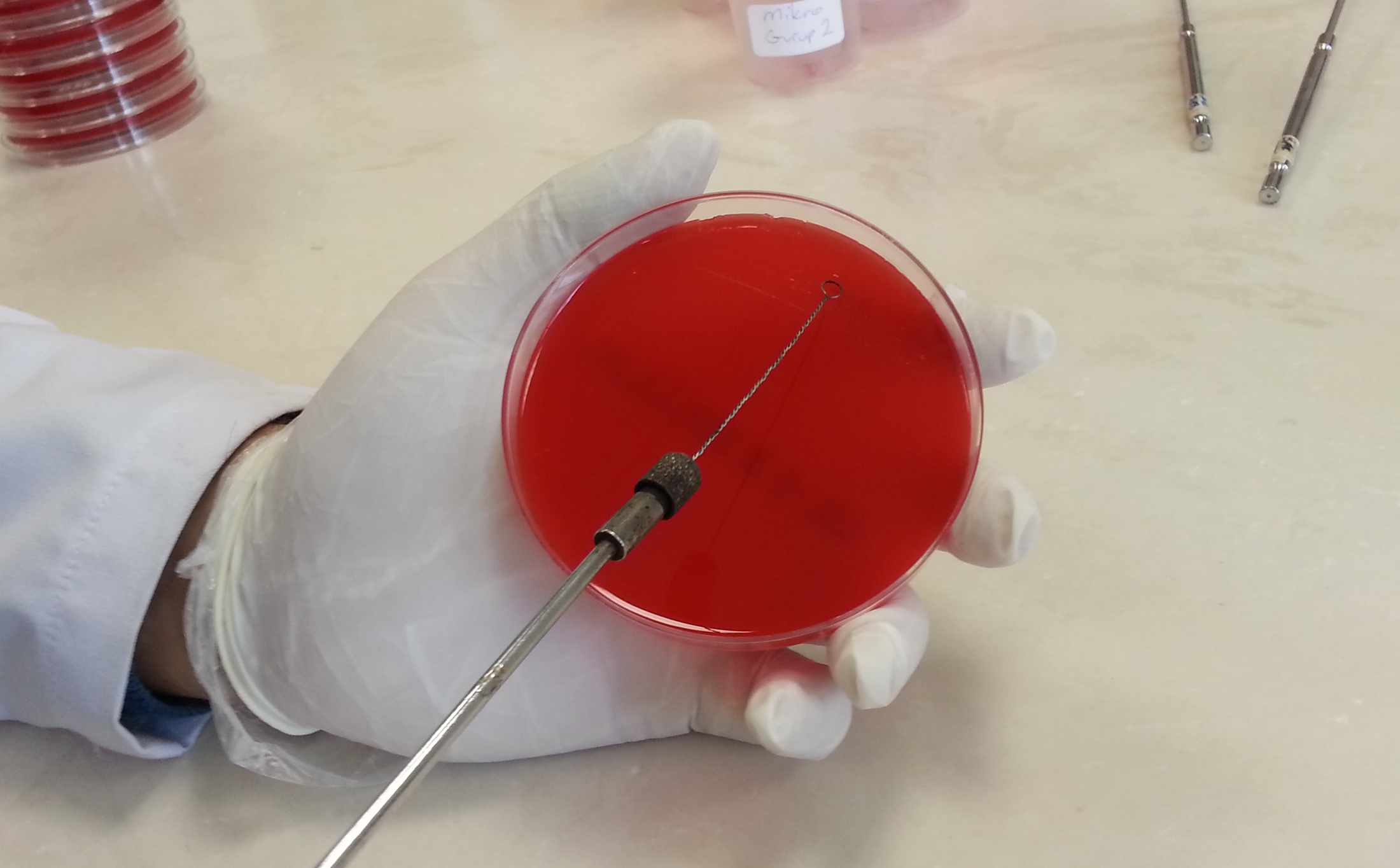

Supplement: S5 Fig — (TIF) [file pone.0165544.s005.tif]
